# Four distinct cytoplasmic structures generate and release specific vesicles, thus opening the way to intercellular communication

**DOI:** 10.20517/evcna.2023.03

**Published:** 2023-03-15

**Authors:** Gabriella Racchetti, Jacopo Meldolesi

**Affiliations:** ^1^San Raffaele Institute, Vita-Salute San Raffaele University, Milan 20132, Italy.; ^2^CNR Institute of Neuroscience at the Milano-Bicocca University, Vedano al Lambro, Milan 20854, Italy.

**Keywords:** Autophagosomes, cargo, ectosomes and exosomes, endocytosis, endo-lysosomes, exocytosis, extracellular vesicles (EVs), lysosome storage disorders (LSDs), membrane fusions, microdomains, multivesicular bodies (MVBs), unconventional protein secretion (UPS)

## Abstract

In all cells, generation and release of specific vesicles are the initial steps of back-and-forth intercellular communication. These processes are critical in normal physiology and pathophysiology. Vesicles have particular functions appropriate to their targets. When stimulated, they are released into the extracellular space. Four cytoplasmic membrane-bound structures generate their particular vesicles. Among these structures, multivesicular bodies (MVBs) can accumulate many small vesicles in their lumen; release occurs upon MVB exocytosis. Ectosomes are larger vesicles characterized by their responses and are generated directly and released independently from specific microdomains pre-established in the thickness of the plasma membrane. Most lysosomes do not generate vesicles. However, unique components of a minor form, the endo-lysosome, constitute the third class of structures that release a few vesicles by exocytosis with molecules and structures inducing changes in the extracellular environment. The autophagosome, the fourth structure, releases several heterogeneous vesicles by exocytosis with malformed bio-molecules, assembled structures, and damaged organelles. Interestingly, the frequent interaction of autophagosomes with MVBs and their exosomes contributes to the regulation and intensity of their action. The specificity and function of released vesicles depend on their membranes’ and luminal cargoes’ composition and dynamics. An ongoing investigation of the various vesicles reveals new properties regarding their generation, release, and resulting extracellular processes. The growth of information about structures and their vesicles progressively extends the knowledge base regarding cell communication and contributes to their clinical applications.

## INTRODUCTION

To begin the first review in this Special Issue dealing with intercellular communications, we highlight four intracellular, membrane-bound structures involved in the direct generation of distinct vesicles ultimately released into the extracellular medium. These structures differ, and their vesicles are characterized by distinct properties depending on their nature: origin, generation, intracellular processes and pathways, and discharge. Given these differences, the vesicles from each structure are indicated by a distinct name. In contrast, upon their release, all such vesicles are denoted with the same name, extracellular vesicles (EVs).

Initially, we will cover the discovery of the first two types of membrane-bound structures^[1-5]^. The first, observed initially by electron microscopy, was called by a name still employed, MultiVesicular Body (MVB)^[5]^. The identification of MVBs as endocytic vacuoles, in particular for their luminal generation and accumulation of small vesicles (50-150 nm), occurred about 35 years ago. Initially called intraluminal vesicles, these vesicles become exosomes upon their release into the extracellular space. Since, however, the exosome name has been employed in the scientific community we will employ it in the context of all cellular conditions, from their generation to their release to the extracellular medium, where all released vesicles are called EVs.

The second membrane-bound structure, identified about 35 years ago, comprises several plasma membrane microdomains. The larger vesicles (150-400 nm), grown outwardly, are then released extracellularly from the cell surface. Given their direct extracellular release, the vesicles of our second type are considered independent from the others. For decades, therefore, they were given names unreasonable for larger vesicles: microvesicles, microparticles, shedding microvesicles, and others^[6]^. Currently, such names have been replaced mainly by ectosomes, a name analogous to (but distinct from) exosomes, i.e., the two vesicles end up outside the cells; however, their properties (and thus their names) are different.

The other two cytoplasmic membrane-bound structures involved in communication are specialized forms of two cytoplasmic organelles, lysosomes, and large autophagosomes. Upon their discovery over 60 and 50 years ago, respectively, these organelles were believed to carry out only a few functions, some dependent on their fusion: uptake, digestion, and elimination of cytoplasmic molecules and structures. Additional functions of these organelles, due to their exocytoses followed by extracellular discharge of cargo components, were discovered approximately 20 and 10 years ago, respectively^[7-10]^.

Regarding dynamic properties, including release and navigation through the extracellular medium, two essential processes occur in the four specific intracellular membrane-bound structures. First, they often respond to stimulation, for example, activation of receptors or stressful conditions; second, they mediate other fusions (usually called “prefusions”) with vesicles or cisternae of the endocytic system, which are the structures taken up by all cells to equilibrate surface enlargements dependent on exocytoses and other processes^[11,12]^. Prefusion with endocytic components is essential for intracellular cytoplasmic structures to perform additional fusions with other cytoplasmic membranes. Studies in the last decade have demonstrated that the various types of membranes need to have experienced prefusions with endosome structures^[11,12]^. Our knowledge of the four membrane-bound structures shows this to be their case. MVBs and their exosomes are largely endocytic. They originate from endocytic cisternae. Ectosomes are generated from plasma membrane microdomains, including endocytic steps, as shown by specific markers^[5,6]^. Lysosomes competent for exocytosis, officially called endo-lysosomes, are generated by their fusion with early-generated endocytic structures^[7,8]^. Competent autophagosome fractions are activated by prefusion with endocytic vesicles during maturation^[9,10]^.

In the present review, we intend to illustrate the role of the four intracellular membrane-bound structures, from their generation to the discharge of their vesicles, including all steps before extracellular navigation. The properties and functions of one type of these structures will be illustrated in a separate section, from numbers two to five. In contrast, endocytic structures are not presented in a single section. The endosomes are included in the corresponding sections.

## MVBs AND THEIR EXOSOMES

In the introduction, we presented MVB, an endocytic cisterna that, by the inward budding and pinching off of its small membrane domains, induces the generation and luminal accumulation of small vesicles, the exosomes. This section is organized into four subsections. The first (2A) is focused on the properties and functions of the whole MVB and has substantial relevance for cell function. Two subsections (2B and 2C) illustrate the generation and properties of specific exocytic vesicle membranes and luminal cargoes. The processes of MVB occurring upon the accumulation of their vesicles (i.e., their intracellular traffic leading some toward the plasma membrane) and their exocytoses, are reported in subsection 2D. The role of MVB and exosomes in diseases, especially neurodegenerative and cancers, have been illustrated in several reviews, including ours^[13,14]^. Therefore diseases are not presented in this review.

**Subsection 2A: MVBs as a whole.** Exosome loading within MVBs depends on sirtuin2, a deacetylase enzyme that participates in several other processes, including protecting neurons from neurodegeneration and stimulating the viability of cancer cells^[15]^. During and upon their vesiculation, MVBs undergo maturation. They move within cells with substantial accumulation in the microtubule organization center. From there, they move alternatively in two directions. Upon interaction with the Rab7 ortholog Ypt2 and the multi-subunit tethering complex HOPS, some MVBs proceed to specific fusion with lysosomes governed by the Qa-SNARE Pep12 protein. Upon such fusion (which accounts for a significant fraction of the MVBs present in the cell), exosomes are discharged into the lysosome lumen, then disassembled, and its components are exposed to catabolism. These exosomes will never be converted into EVs^[16]^.

**Figure 1:**
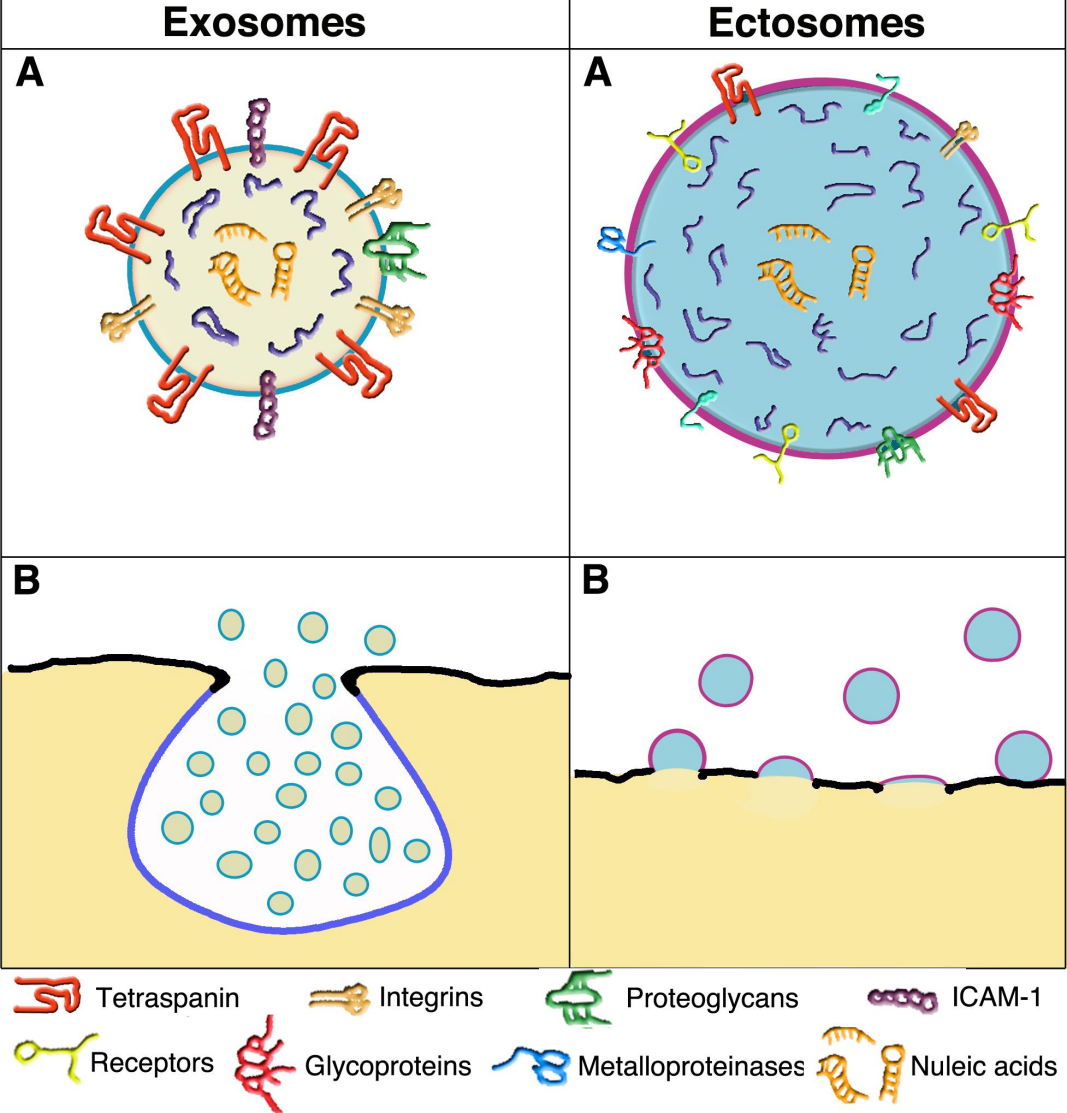
Structure and composition of the two vesicles; their release from the plasma membrane by exocytosis and outward budding. A comparison of the images confirms that exosome vesicles are smaller than ectosome vesicles. Plasma membranes of exosomes are in black, whereas those of vesicle membranes are different: sky blue for those of endocytic nature, i.e., the MVB (B) and its exosomes (A and B); violet for those of ectosomes (A and B), in which plasma membrane and endosome are mixed. In exosome and ectosome vesicles, the color of the lumen is substantially different: lemon yellow for exosomes (A and B) and maya blue for ectosomes (A and B). The present comparison emphasizes the moderate chemical distinction between the vesicle types (A). Several essential membrane proteins are listed below. Some (e.g., tetraspanins and integrins) predominate in exosome but are also present in ectosome. In contrast, other proteins (e.g., receptors, glycoproteins, and metalloproteinases) are present in ectosomes and are not appreciable in the exosome. In both A vesicles, nucleic acids are present (neon orange) in the depth of the lumen. B images show the release of the two vesicle types: by exocytosis of MVBs, with the subsequent release of exosomes, by outward budding of ectosomes followed by growth and then outward release from the plasma membrane. The images of the two enlarged vesicles shown in two A are reproduced with permission^[26]^.

The remaining MVBs undergo exocytosis [Figure 1]. When lysosomes are disrupted, the alternative exocytoses are significantly increased^[17-19]^. The initial step of the process is the MVB movement toward the cell surface, increased by stimulatory signals. MVBs include a form of cooperation established with another structure of vesicle generation, the autophagosomes. Comparative studies with and without autophagy inhibitors have revealed the importance of this process. When autophagosomes are unavailable, exocytoses of MVBs are significantly reduced; when autophagosomes are abundant, exocytoses are increased^[20]^. The integration of these two structures (illustrated in detail in subsections 5B and 5D on autophagosomes) regulates some form of MVB exocytosis.

**Subsection 2B: Exosome membranes **[Figure 1]**. **The exosome membranes contain phospholipids and lysobisphosphatidic acid (LBPA), an atypical phospholipid absent in many other types of membranes, cholesterol and ceramide^[21]^. The tetraspanins are abundant (in various forms, predominantly CD63) and critically crucial for exosome assembly. Also essential are the integrins that convert signals across the vesicle membrane and other proteins at a lower concentration, including adhesion proteins, receptors, glycoproteins, and metalloproteases [Figure 1]^[21]^.

To induce their generation and curvature, exosomes operate with associated protein complexes, i.e., ESCRT-0, -I, and -II^[22]^. ESCRT-III subunits, with helical filaments of various forms, mediate membrane remodeling by interacting with vacuolar protein sorting (VPS) and an ATPase^[22-25]^. Fission occurs when ESCRT-III is removed, leading to complete separation of the exocytic neck and ensuing exosome formation within the MVB lumen [Figure 1].

**Subsection 2C: Exosome cargoes **[Figure 1]**.** The accumulations of cytoplasm within small initial MVB protrusions begin cargo growth. Some cytosolic proteins are typical of these cargoes. Its surface proteins are often anchored (by myristoylation, palmitoylation, or other sequences) to the luminal membrane surface of exosomes. Other proteins, including the ESCRT-associated TSG 101, ALIX, and some HSPs, participate in post-translational modifications and complex assembly^[21,27]^, contributing to cargo growth^[27-29]^. Proteins are not the only components of cargo, which contain molecules of different natures. There are also small sequences of DNA, lipids, and metabolic molecules; highly abundant are various types of RNA (mostly microRNAs, miRs, together with messenger RNAs, long non-coding RNAs, and ribosome RNAs). The cargo molecule presence is relevant. In fact, RNA-binding proteins undergo condensation^[25,27]^. Together with essential factors such as IL-1β and TNF-α, various proteins undergo selective engulfment within exosomes^[30,31]^. In addition, recent results have confirmed the relevance of protein-RNA binding. Condensed YBX1 proteins induce liquid-liquid phase separations in cargoes, recruiting miR-223 and enabling their targeting and packaging within growing exosomes^[31]^. Further details have been clarified by the identification, active in the assembly of cargoes, of proteins containing endofin (i.e., a protein domain confined to endosomes, and of ARRDC1, an adapter of ubiquitin ligases)^[30,32]^. Moreover, vesicle cargoes accumulate additional factors by a transport process dependent on another protein, LAMP2A^[33]^. Finally, the interest in cargoes should also be focused on unconventional secretion processes (UPS). Many involved proteins are loaded into exosomes^[34-36]^.

**Subsection 2D: Journey of MVBs and exosomes**. Once heavily loaded by numerous exosomes, MVBs travel within the cell. In response to various types of stimulation they move, approaching the plasma membrane. Upon tethering to specific sites^[37]^, there is some heterogeneity of the molecules participating in MVB exocytosis. The first Ras GTPase active in the process is Ras11. Additional GTPase forms, such as Rab27a and Rab 27b and members of the Rho, Rac, and cdc42 family, have been reported to operate in many, but not all, cell types^[38,39]^.

Concerning exocytic fusion, the protein most frequently involved in various tissues and cancers is R-SNARE VAMP7 with Q-SNARE SNAP23^[40,41]^. However, other R-SNAREs (including VAMP3 and VAMP8) are also effective with lower frequency^[42,43]^. Their ternary complex, established with SNAP23 associated with syntaxin-4, induces the generation of enlarging pores, called invadopodia, critical sites for MVB fusion with the plasma membrane and the subsequent exosome release^[40-43]^. Small GTPases include Ral, Rab (especially Rab35), and other Ras^[44]^. The integrated analysis of the various participants has revealed the role of non-coding RNAs and G protein-coupled receptors^[43,44]^. The latter, via their cAMP effect, promote the fusion via a SNAP23 phosphorylation at the Ser 110 position^[43]^.

Recent developments in pH-dependent fluorescence microscopy revealed exocytoses’ frequency, localization, and machinery. Exosome localization experiments have revealed unexpected results. In lymphocytes, the site of exocytosis is redistributed upon the establishment of immune synapses^[35]^. In epithelial cells, MVB exocytoses addressed to the basolateral area differ from those addressed to the apical area. Differences have also been demonstrated between the two corresponding families of released exosomes^[39,45]^. Therefore, heterogeneity is a common property of exosomes discharged even by single cells. Finally, recent evidence has demonstrated that the release and distribution of exosomes in the brain plays an unexpected, critical role in the pathogenesis of neurodegenerative diseases, a new role that will be investigated in the near future^[46]^.

## ECTOSOMES WITH THEIR SHORT INTRACELLULAR LIFE

Knowledge of ectosomes (more limited than exosomes) has been questioned for many years. Ectosome fractions, recovered from EV mixtures, have been found in lower numbers (sometimes much lower) than exosomes^[5,6,31,46]^. However, in a few studies, the fractions of the two vesicles are close^[47,48]^. The prevalence of exosomes is, therefore, frequent; however, it is not the rule. Therefore, the comparison of the two vesicles should be considered (and possibly established by advanced techniques) in EV mixtures investigated in various conditions.

During the past decade, information about ectosomes has increased significantly. The mechanisms of their generation differ substantially from those of the other vesicles, e.g., there are no exocytoses such as those of the other membrane-bound structures. Instead, there are outward budding and vesicle release from the external surface of the plasma membrane to the extracellular space [Figure 1]. Interestingly, the microdomains involved in ectosome generation are not always spread in flat plasma membrane areas. Some are concentrated over cell protrusions, such as filopodia and microvilli, operating as specialized platforms for vesicle budding^[49]^. Even if these processes are particular, some pre-EV properties (subsection 3A), i.e., the generation of the vesicle membranes (subsection 3B) and the assembly of their cargoes (subsection 3C), are somewhat analogous to those of exosomes presented in section 2. Also analogous is our decision to omit the role of ectosomes in diseases from this review. This decision has already been discussed in section 2 regarding exosomes^[13,14]^.

**Subsection 3A: Intracellular life.** The life of ectosomes is much shorter than that of other vesicles. Intracellular ectosomes exist only during their generation, growth, and release; then, they are considered among the EVs. Their plasma membrane microdomains, i.e., the sites of ectosome generation, appear different from the rest of the plasma membrane. For example, the asymmetric phospholipid layers of their membrane are rapidly rearranged. Several membrane proteins are analogous (however not identical) to those of exosomes. For example, the most abundant tetraspanin of ectosomes is CD9^[48]^, not CD63, which predominates in exosomes^[21,48]^. Proteins typical of the plasma membrane are present in ectosome membranes during and after their generation; however only at low concentrations^[50]^. In cells stimulated by appropriate receptor agents (such as ATP), ectosome generation/release starts within the first few minutes^[50,51]^. Cdc42 is a small G protein of the Rho family and a convergent node of multiple regulatory signals. The binding to its downstream effector Ras GTPase-activating-like protein 1 is required for ectosome shedding^[52,53]^. Additional stimulatory events sustained by LINK1 (a kinase that controls the dynamics of actin cytoskeleton) are upregulated by RhoA and Rock. Inhibitors of these G proteins suppress the production of ectosomes^[53,54]^. Subsequent developments have been confirmed by the involvement of ESCRTs and their associated proteins^[54,55]^ analogous (but not identical) to those of exosomes reported in Subsection 2A.

**Subsection 3B: Ectosome membranes **[Figure 1]. Two primary membrane processes activate typical ectosome generations. Upon establishing plasma membrane microdomains, the first involves their dynamics, with ensuing outward budding and fission^[54,55] ^[Figure 1]; the second is based on the ESCRT-III complex, followed by the appropriate ATPase, governing the increasing curvature of growing vesicles. The ensuing narrowing of their neck, followed by their final scission, is followed rapidly by the release that converts ectosomes into released EVs^[48]^. These and other processes of membrane regulation include various processes such as protein phosphorylation and calmodulin activation^[56]^. Based on its properties, the intracellular life of ectosomes appears to play a critical role in the regulation of vesicle biology^[57]^.

**Subsection 3C: Ectosome cargoes **[Figure 1]. Knowledge about ectosome cargoes is limited. Their accumulation of proteins with high affinity binding to micro RNAs is similar to exosomes^[28,31]^ (subsection 2C). Among such proteins is ARRDC1, a ubiquitin ligase adapter that regulates ectosome generation and release^[58]^. Loading of ectosome cargoes by RNA-binding proteins and miRNAs is supported by the LC3-conjugated machinery, an example of vesicle/autophagy interaction^[59]^. The ectosome cargo formation requires some regulation by caveolin-1, a structural protein typical of plasma membrane caveolae, which is also abundant in ectosomes; in contrast, caveolin-1 is not abundant in exosome cargoes^[56]^. Additional components of ectosome cargoes are IL-1β and other cytokines and factors of the TNF family with proteins destined to be secreted by UPS^[60]^. Upon their release from the plasma membrane, the ectosomes, not yet fully distinguished from the other EVs, remain associated with the cell's surface (however, for short times only).

## NEWLY-DISCOVERED LYSOSOME FUNCTIONS

As already anticipated in the introduction, for decades the only function recognized for lysosomes was passive digestion induced by their uptake of large numbers of molecules and organelles. In recent years, additional specialized functions have been discovered and characterized, governed by their cooperation with members of the endocytic system mediated by the Rab11 GTPases^[61]^. These results have demonstrated the multiplicity and complexity of lysosomal functions^[7]^. These properties will be presented in three subsections: 4A, covering endo-lysosomes; 4B, dealing with cargoes; and 4C, covering lysosomal disorders.

**Figure 2:**
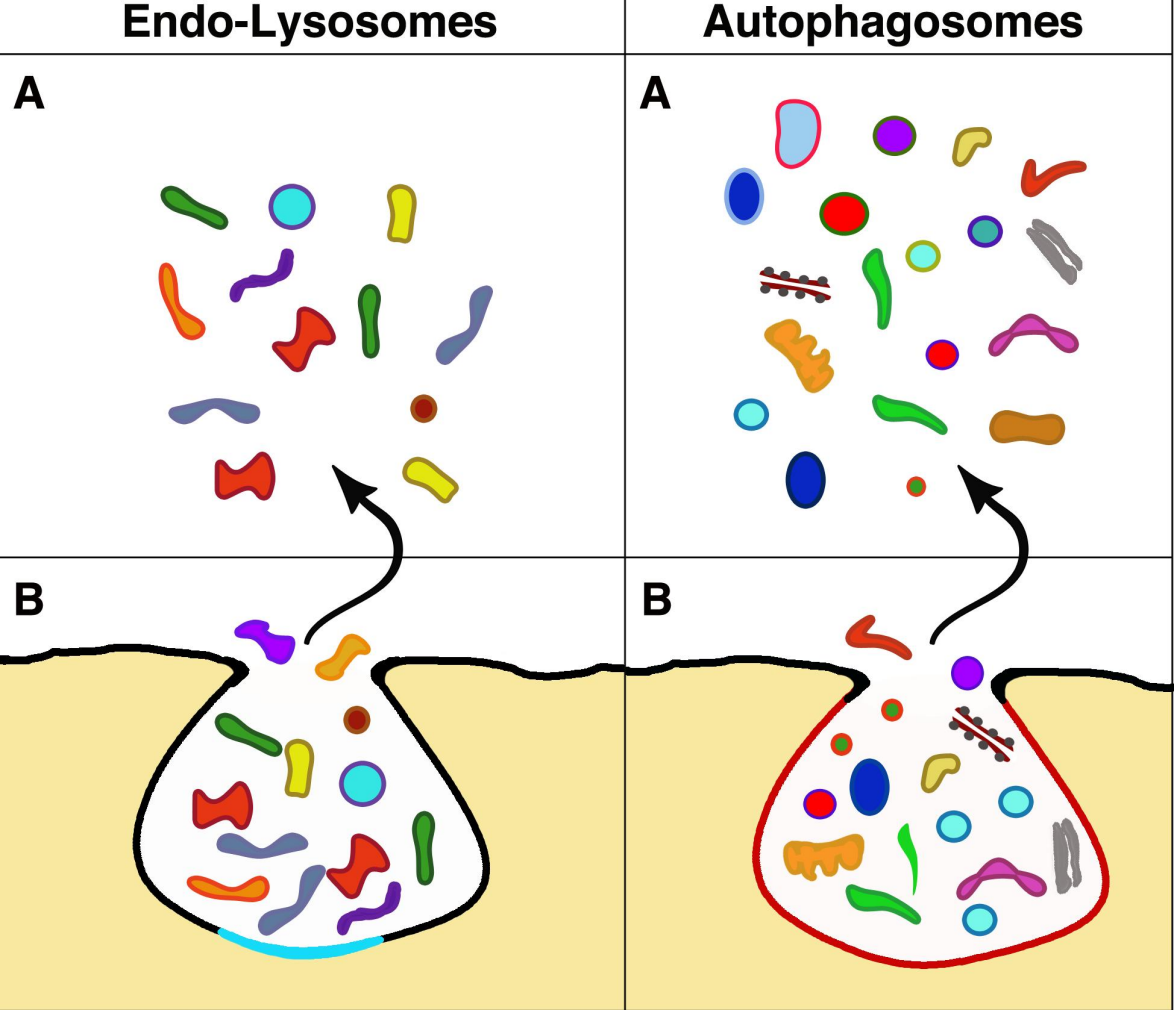
Exocytosis of the two organelles (B) and their released material spread in the extracellular space (A). Analogous to the exosomes in Figure 1, these images of an endo-lysosome and an autophagosome illustrate exocytoses with release to the extracellular space. However, the two exocytosis forms differ concerning exosomes in Figure 1 and from one another. The endo-lysosome B image is delimited by a membrane combination induced by the fusion of a lysosome (black, the same color as the plasma membrane) with an endocytic cisterna (sky blue). The other B comes from a mature autophagosome with a membrane drawn in a candy color. The discharged cargoes (A) of endo-lysosome includes various types of enzyme molecules with degraded organelles, structures, and very few vesicles. The A image of the autophagosome shows some preservation of various structures, including cytoplasmic organelles (mitochondrion, endoplasmic reticulum, Golgi complex) and a significant number of vesicles, variable in size, color, and membrane, likely originated from the autophagy of various cytoplasmic structures.

**Subsection 4A: The endo-lysosomes.** The new identification of lysosomal functions emerged unexpectedly upon the discovery of endo-lysosomes, a form of organelle fused with an early endocytic structure of considerable surface extension^[62]^. The investigation of this innovative form of lysosomes revealed the co-expression of properties typical of endosomes, including various forms of flotillin, several cargo proteins, the TPC2 cation channels, and others^[63-66]^. Several functions have been reported to depend on endo-lysosomes. The first were nutrient sensing, intracellular signaling, and intracellular metabolism^[61,62]^. More recently, endo-lysosomes revealed additional processes such as intracellular trafficking, lysosome pH regulation, fusion/fission with other organelles, and examples of cell secretion^[63-66]^. For the present review, the endo-lysosome function of highest interest is exocytosis established by fusion with the plasma membrane [Figure 2]. Markers of this process can be the appearance at the cell surface of luminal lysosomal epitopes such as LAMP1^[67-69]^, followed by an endocytic recycling response, activated to compensate the excess of surface space/composition increase induced by the endo-lysosome exocytosis^[68,69]^. The latter is a Ca^2+^-dependent process controlled by the G proteins Rab11a and Rab11b, oriented toward the plasma membrane. Rab11s interact with other molecules, such as the guanine nucleotide exchange factor GRAB and Rab3a^[61,67]^. Thus, endo-lysosome exocytosis is a precisely controlled process.

**Subsection 4B: Endo-lysosome cargoes** [Figure 2]. A critical aspect of endo-lysosome exocytosis is the nature of cargo components discharged extracellularly^[63,68]^. Within lysosomes, the structures are at least partially digested. However, some conserved complexes, such as tetrameric and heterodimeric structures, resist digestion^[63]^. Enzyme release (in particular of a proteomic nature) can affect the structure of the extracellular matrices. In addition, endo-lysosome exocytoses have been reported to release vesicles, including some accumulated upon previous fusions with MVBs and autophagy [Figure 2]. Therefore, there is a chance for endo-lysosome exocytosis releasing a small fraction of operative EVs^[63,68]^.

**Subsection 4C: Effects on other organelles induced by lysosome disorders.** A final question about lysosomes deals with its lysosome storage disorders (LSDs) induced by their alterations in neurodegenerative diseases and some cancers. The mechanisms involved in generating this pathology vary, including increased fusion with MVBs and the subsequent release of exosomes. These findings suggest that exosomes loaded with various medications can be considered for therapy^[20,70,71]^. LSDs are characterized by accumulating wholly or partially undigested cellular waste intrinsic to each disorder^[70-73]^. In other cases, the defect occurs via an excess of endo-lysosomal ion channels, altered by interaction with cholesterol and lactosylceramide^[70]^. Several lines of evidence suggest that lysosomal dysfunction induces distinct MVB exocytoses that participate in the pathogenesis of LSD diseases and may be involved in the development of their therapy^[7,20,68,74]^. Analogously, during lysosome inhibition, several steps are required in the formation and secretion of classical autophagosomes by the small GTPase Rab27A^[75]^.

## AUTOPHAGOSOMES

Autophagosomes are organelles developed by cells during starvation under the control of the small G protein Rab37^[75]^. Their task is to maintain homeostasis by providing nutrients from structures and molecules, including damaged organelles and malformed bio-macromolecule, all removed from the functional cytoplasm. Initially, autophagosomes are assembled by the growth of *de-novo*-formed double membranes. Autophagosome persistence within the cells includes the integration of markers, fusion with endosome components, and maturation completion^[75,76]^. Accurately regulated processes (not shown in Figure 2) include the final steps of autophagosome interactions with MVB and exosomes. Moreover, autophagosomes often fuse with lysosomes, and the ensuing mixed organelles are called autolysosomes. Upon such fusion, the cargoes of autophagosomes are degraded. Their fragments are recycled via specific pathways^[77-79]^.

Autophagy induces primarily protective and recycling effects. To confer adaptation to the ever-changing environments, their interactions and fusions need to be tightly regulated. It is clear that various human pathologies deregulate autophagy, and its modulations have significant therapeutic potential^[80,81]^. Three forms of autophagosome exist, distinguished by their different size. The one operative in intercellular communication is the large form called the macroautophagosome. Given its unique function considered in the present review, the active macroautophagosome form is called by the general name of the autophagosome. The properties of the latter are summarized in 5A, traffic, and exocytoses, direct and mediated via endo-lysosomes; 5B, the interactions established by autophagosomes with MVBs and exosomes; 5C, the establishment and regulation of protein secretion; and 5D, the effects of autophagosomes in various diseases, especially in cancers.

**Subsection 5A: Autophagosome trafficking and exocytoses. **The autophagic machinery, adapted to unable protein trafficking and UPS secretion^[20,75,76]^, operates with the expression of family markers such as LC3-II, SQSTM1/p62, and core autophagy (ATG) proteins^[75,76]^. Of these markers, ATG9A concentrates in a compartment comprising clusters of vesicles and tubules^[9]^ and participates in the movement of cell lines. The pH-fluorine labeling technique revealed that ATG9A is distributed toward the migration front, with protrusive activity triggered with clathrin adapter complexes. Therefore, ATG9A governs vesicular trafficking, allowing the expansion of cell protrusion toward the extracellular matrix^[82]^.

Autophagic exocytoses have been confirmed by the demonstration of specific markers among EVs^[75-77,82]^. Moreover, the primary functions of autophagosomes, including their participation in cancers and UPS secretion, discussed in the following subsections 5C and 5D, imply the existence of autophagosome cargoes of exocytic vesicles released to the extracellular space^[9,20,77] ^[Figure 2]. The properties and the details of autophagocytic exocytosis, with the involvement of the small GTPase Rab27a and the SNARE protein Sec22b, have been characterized several times^[76]^. However, autophagic exocytosis needs to be further investigated to circumvent the limitations of previous studies. An additional process, resulting in the release of autophagic markers, could be due to the prefusion of autophagosomes with endo-lysosomes, the form of lysosomes competent for exocytosis. Exocytoses of autophagosome/endo-lysosome fusions have been reported^[82-85]^. Therefore, although limited in extent, the contribution of this indirect exocytosis^[76,85]^ cannot be excluded.

**Subsection 5B: Autophagosome interactions with MVBs and exosomes. **Alternatively to the direct fusion with lysosomes, autophagosomes can establish interactions with MVBs named amphisomes^[20]^ (mentioned in subsection 2A). The autophagosome/MVB interactions, established in the proximity of the plasma membrane, are significant. They increase the frequency of MVB exocytoses, which in contrast are decreased by the removal of autophagosomes and by their inhibitors^[20,86,87]^. This cooperation occurs in many types of cells, including some plants^[88]^.

In addition to their interaction with MVB, autophagosomes interact with their established vesicles, the exosomes. The process of this interaction is highly complex; it could be established in target cells, where exosomes of original cells are taken up, or in the extracellular space, where exosomes and autophagosomes are present as EVs^[72,89,90]^. Autophagy and exosome crosstalk are complex processes. They are beginning to be recognized and documented^[86,89-92]^. These interactions, considered protective of cells, including neurons^[80]^, have been shown to play an essential role in cancers, discussed in the following subsection 5D.

**Subsection 5C: Secretory autophagy. **Autophogasome secretion occurs by UPS. Processes of this type are also frequent with exosomes, ectosomes, and lysosomes^[7,34-36]^. With autophagosomes that govern many cytoplasmic proteins, UPS is critical, leading to toxic protein disposal, immune signaling, and pathogen surveillance. For decades UPS was the only type of secretion active in autophagosomes for decades. Recently, based on their endoplasmic reticulum-translocation process, about 30% of the autophagy-dependent proteins have been reported to be possibly secreted by conventional processes^[93]^. In other words, many (but not all) secreted autophagic proteins operate by UPS, while a smaller fraction appears to be operated by conventional forms of secretion^[93,94]^. Various proteins secreted by UPS have been identified. Some participate in the extracellular navigation of EVs, which is essential for various functions^[20,75,76,85]^. Among autophagosome EVs, many contain cytokines, granules (including FGF2, TGFβ), and proteins (α-synuclein, HMGB1, matrix metalloproteases, and many others)^[89,95,96]^, also relevant for diseases as discussed in the subsection 5D.

**Subsection 5D: Autophagosome diseases. **The role of autophagosomes in diseases has been reported in various organs, for example, cerebral neurodegeneration^[97]^. For immune diseases, autophagosomes have been investigated more extensively; however, the state of knowledge remains limited^[98]^. Regarding therapy, the studies of autophagosome EVs are preliminary^[80,81,92]^.

More established is the state of cancer. A consistent property of cancer cells is their substantial generation of exosomes and (thus) of their EVs, which interact extensively with autophagosomes, contributing to some forms of cancer. Specifically, the exosomes secreted by cancer cells modulate autophagy in recipient cells, while autophagy influences exosome biogenesis^[89,91]^. The promising approach is based on the anti-cancer effects induced by autophagy inhibitors, which significantly impact in terms of EV quantity and their content, with profound therapeutic activity^[20,99,100]^. Reinforcement of autophagosome effects can be induced by agents such as nuclear factors and G protein-coupled receptors^[10,101]^. Therefore, interest is currently focused on developing inhibitors of various properties. Results appear based on the interaction of autophagosomes with exosomes and other factors that, in many cancers, are essential for adapting patients to tumor microenvironments^[20,89,99-101]^.

Another mechanism that controls cancer growth is a property typical of endo-lysosomes reported in subsection 4A, i.e., the expression of TRP channels. These channels, absent from general lysosomes, are often maintained by the endo-lysosomes fused to autophagosomes, with or without MVBs. Therefore, cancer growth can be reduced by specific anti-channel treatments^[65,102]^. In this case, the therapy can be attempted based on current autophagosome knowledge.

## CONCLUSION

We illustrated the initial steps of vesicle processes leading to EV navigations and intercellular communications. Most illustrations concern cytoplasmic areas where specific vesicles are generated. For many years, no function had been attributed to the original membrane-bound structures of vesicle origin. The discovery of the first two types of specific structures, MVBs (visible in all cells) and ectosomes (assembled at the plasma membrane and then pinched off) have revealed mechanistically innovative vesicle generation relevant to cellular physiology and pathology. Recently, two additional types of structures (lysosomes and autophagosomes, already well known for their single intracellular functions) were recognized to participate in vesicle generation. The entire process, therefore, is due to four types of cytoplasmic structures characterized by distinct properties, interconnected by various functions, including fusions, coordination with the exchange of molecules, and differential effects induced by their function.

Four distinct structures might appear too many to start governing vesicles and their EV generations with subsequent intercellular communications. It should be considered, however, that these structures and their products are substantially different. In most cells, MVBs and their exosomes predominate in numbers and functions. Ectosomes (larger vesicles that is discharged faster) are the only ones directly assembled and released from the plasma membrane. Endo-lysosome cargoes contain only a few vesicles with many essential molecules and enzymes involved in extracellular protein turnover. Autophagosome function is precisely regulated. With various structures and molecules, they release heterologous vesicles that remain to be characterized in future analyses^[40,90,103]^. The properties of the four structures and their products have opened the way to intercellular communications. In addition to their scientific progress, they have substantial medical relevance, already presented by a vast body of literature.

## DECLARATIONS

### Availability of data and materials

The data were obtained from PubMed, NCBI, USA, and our Institute’s and University’s libraries.

### Copyright

© The Author(s) 2023.
